# Comparative transcriptome analysis revealing dormant conidia and germination associated genes in *Aspergillus* species: an essential role for AtfA in conidial dormancy

**DOI:** 10.1186/s12864-016-2689-z

**Published:** 2016-05-17

**Authors:** Daisuke Hagiwara, Hiroki Takahashi, Yoko Kusuya, Susumu Kawamoto, Katsuhiko Kamei, Tohru Gonoi

**Affiliations:** Medical Mycology Research Center (MMRC), Chiba University, 1-8-1 Inohana, Chuo-ku, Chiba, 260-8673 Japan; Molecular Chirality Research Center, Chiba University, 1-33 Yayoi-cho, Inage-ku, Chiba, 263-8522 Japan

**Keywords:** Conidia, Dormancy, Germination, Aspergillus, AtfA, Transcriptome

## Abstract

**Background:**

Fungal conidia are usually dormant unless the extracellular conditions are right for germination. Despite the importance of dormancy, little is known about the molecular mechanism underlying entry to, maintenance of, and exit from dormancy. To gain comprehensive and inter-species insights, transcriptome analyses were conducted across *Aspergillus fumigatus*, *Aspergillus niger*, and *Aspergillus oryzae*.

**Results:**

We found transcripts of 687, 694, and 812 genes were enriched in the resting conidia compared with hyphae in *A. fumigatus*, *A. niger*, and *A. oryzae*, respectively (conidia-associated genes). Similarly, transcripts of 766, 1,241, and 749 genes were increased in the 1 h-cultured conidia compared with the resting conidia (germination-associated genes). Among the three *Aspergillus* species, we identified orthologous 6,172 genes, 91 and 391 of which are common conidia- and germination-associated genes, respectively. A variety of stress-related genes, including the catalase genes, were found in the common conidia-associated gene set, and ribosome-related genes were significantly enriched among the germination-associated genes. Among the germination-associated genes, we found that *calA*-family genes encoding a thaumatin-like protein were extraordinary expressed in early germination stage in all *Aspergillus* species tested here. In *A. fumigatus* 63 % of the common conidia-associated genes were expressed in a bZIP-type transcriptional regulator AtfA-dependent manner, indicating that AtfA plays a pivotal role in the maintenance of resting conidial physiology. Unexpectedly, the precocious expression of the germination-associated *calA* and an abnormal metabolic activity were detected in the resting conidia of the *atfA* mutant, suggesting that AtfA was involved in the retention of conidial dormancy.

**Conclusions:**

A comparison among transcriptomes of hyphae, resting conidia, and 1 h-grown conidia in the three *Aspergillus* species revealed likely common factors involved in conidial dormancy. AtfA positively regulates conidial stress-related genes and negatively mediates the gene expressions related to germination, suggesting a major role for AtfA in *Aspergillus* conidial dormancy.

**Electronic supplementary material:**

The online version of this article (doi:10.1186/s12864-016-2689-z) contains supplementary material, which is available to authorized users.

## Background

Conidia are, in general, stress-tolerant reproductive structures, and filamentous fungi vigorously produce conidia under the appropriate conditions [1]. In the presence of water and appropriate nutrients, conidia germinate, whereas conidia enter dormancy when the environment is not appropriate. Dormant conidia are metabolically inactive and are viable for a long time (more than a year) [2]. Therefore, dormant conidia do not consume energy prior to encountering the appropriate conditions for germination. This mechanism allows conidia to find an environment where the fungi can prosper, which consequently contributes to their ubiquity and prosperity in nature. Despite its significance to conidial physiology, however, the molecular mechanisms underlying entering and exiting dormancy remain largely unknown.

To gain insights into dormancy mechanisms, genes and proteins that are specifically highly expressed in conidia, or during germination, have been investigated by transcriptomic or proteomic approaches in this decade. Van Leeuwen et al. [3] found that 4,628 out of 14,253 genes were expressed in *Aspergillus niger* dormant conidia using Affymetrix microarray chips. Additionally, more than half of the genes changed their expression levels at the beginning of the early germination stage (~2 h). Novodvorska et al. [4] showed that 6,519 genes (42.3 %) were differentially expressed [> twofold fragments per kilobase of transcript per million mapped reads (FPKM)] during the first hour of germination in *A. niger*, as detected by an RNA-sequencing analysis. Of these, 2,626 genes had increased expression levels, and functional categories related to RNA processing, protein synthesis, and nitrogen metabolism were enriched in the gene set. Proteome analyses regarding *Aspergillus* conidia were reported by two groups in 2010. Teutschbein et al. [5] presented a proteome of *Aspergillus fumigatus’* resting conidia. They detected 449 proteins [4.7 % of predicted open reading frames (ORFs)] in the conidia and 57 were overrepresented compared with in hyphae. Interestingly, pyruvate decarboxylase and alcohol dehydrogenase were found in dormant conidia, suggesting that alcoholic fermentation might occur during dormancy. They also revealed the presence of several *A. fumigatus* allergens, including Asp f3, Asp f13, Asp f22, Asp f27, and CatA in the conidia. However, Oh et al. [6] investigated the *Aspergillus nidulans* proteome 1 h after germination and 144 proteins were found to be differentially expressed. These two proteomic data partly overlapped the data from the transcriptomic analysis, even though they were performed using different *Aspergillus* species under different culture conditions.

Genome sequence data with annotations for four representatives of the *Aspergillus* species (*A. nidulans*, *A. fumigatus*, *A. niger*, and *Aspergillus oryzae*) are now available from Aspergillus genome database, AspGD (http://www.aspgd.org/), which is advantageous to the investigation of the universality and diversity of the intra-genus genomes. Indeed, a comparative genome analysis was conducted and provided a vast amount of information on the biology and physiology of filamentous fungi [7–9]. However, studies comparing different species at the transcriptional level have not been undertaken. Because cost-effective RNA-sequencing technology is available, we are now able to determine the transcriptomes of different *Aspergillus* species and make inter-species comparisons, which could provide new findings.

In the present study, we mainly addressed the issue of dormancy mechanisms in *Aspergillus* conidia by performing a comparative transcriptome analysis within the genus. Indeed, we focused on the conidia and 1 h-grown conidia of *A. fumigatus*, *A. niger*, and *A. oryzae* for our comparative transcriptome analysis. First, we defined the genes that were dominantly expressed in conidia or germinating conidia as conidia-associated genes (CAGs) or germination-associated genes (GeAGs), respectively. Subsequent comparisons of the CAGs or GeAGs among three species exposed the common conidial features of these filamentous fungi.

## Results

### Transcriptome determination using RNA-sequencing

To compare the conidial transcriptomes of different *Aspergillus* species, the conidia of each strain should be harvested from physiologically similar cultivation conditions. We chose potato dextrose agar (PDA) and potato dextrose broth (PDB) for the culture media because all of the strains (*A. fumigatus* Af293, *A. niger* IFM 58835, and *A. oryzae* RIB40) produced a large amount of conidia on PDA. Based on the colony expansion rates, conidia production rates, and germination rates (Additional file 1), we grew them at preferred temperature condition, namely *A. fumigatus* were grown at 37 °C, *A. niger* at 30 °C, and *A. oryzae* at 30 °C on PDA.

We first made comparisons among hyphae, dormant conidia, and 1 h-grown conidia in each species. The conidia were harvested from a 7-day-old culture at temperature specified above, and RNA was extracted from the conidia. Similarly, RNA in hyphae was prepared from mycelia cultivated in PDB at the sub-stationary phase according to dry weight (Additional file 1). To get insight into early response of conidia to the condition preferable to germinate, RNA was extracted from the conidia incubated in PDB at the appropriate temperature for 1 h. At the time-point, no germ tubes were appeared at all and the conidia do not start swelling in all species.

Transcriptomes from three different growth phases (hyphae, conidia, and 1 h-grown conidia) of *A. fumigatus*, *A. niger*, and *A. oryzae* were determined by RNA-sequencing analyses. In *A. fumigatus*, 97.8, 96.5, and 95.5 % of the ORFs were expressed in hyphae, conidia, and 1 h-grown conidia, respectively (Table 1). In *A. niger*, 86.7, 83.3, and 72.0 % of ORFs were expressed in hyphae, conidia, and 1 h-grown conidia, respectively, while in *A. oryzae*, 87.0, 85.8, and 77.1 % of ORFs were expressed in hyphae, conidia, and 1 h-grown conidia, respectively. There were 93, 1,251, and 815 genes whose expression levels were not detected in any of the three phases in *A. fumigatus*, *A. niger*, and *A. oryzae*, respectively (Table 1). The mean FPKM for each transcriptome was calculated and 16.3–19.4 %, 12.0–15.3 %, and 11.0–18.8 % of genes showed FPKM values higher than the mean FPKM in *A. fumigatus*, *A. niger*, and *A. oryzae*, respectively (Table 1).

**Table 1:** The numbers of ORFs expressed in each phase of the Aspergillus fungi

		Hyphae					Conidia					1 h-grown conidia				
	# of ORFs	# of expressed ORFs	%	mean FPKM	# > mean	%	# of expressed ORFs	%	mean FPKM	# > mean	%	# of expressed ORFs	%	mean FPKM	# > mean	%
*A. fumigatus*	9783	9569	97.8	69.00	1900	19.4	9445	96.5	87.67	1599	16.3	9341	95.5	91.33	1906	19.5
*A. niger*	14056	12183	86.7	40.83	2146	15.3	11711	83.3	41.82	2083	14.8	10123	72.0	108.12	1682	12.0
*A. oryzae*	11902	10353	87.0	31.79	2234	18.8	10217	85.8	49.97	1948	16.4	9173	77.1	108.5	1313	11.0

### Determination of CAGs and GeAGs

**Fig. 1:**
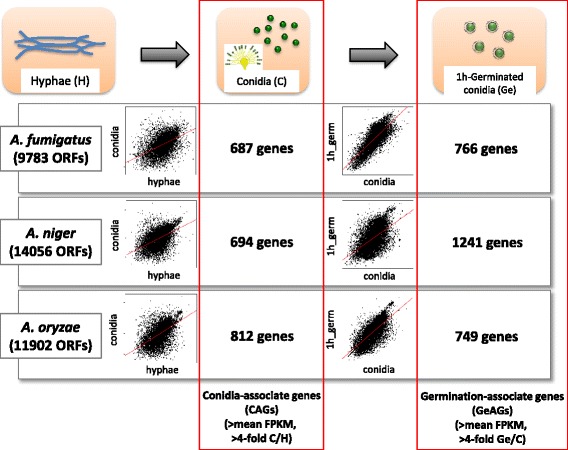
Experimental settings and a summary of the comparative transcriptomic analysis. The conidia-associated genes (CAGs) were determined by comparisons between FPKMs of conidia and hyphae (C/H). The germination-associated genes (GeAGs) were determined by comparisons between FPKMs of 1 h-grown conidia and conidia (Ge/C)

To determine the genes whose expression is enriched in conidia or 1 h-grown conidia, we compared transcriptomes between hyphae and conidia, or between conidia and 1 h-grown conidia, respectively. The CAGs and GeAGs were defined as follows: 1) The expression levels in conidia or 1 h-grown conidia increased more than fourfold compared with that in hyphae or conidia, respectively; and 2) The expression levels in conidia or 1 h-grown conidia conidia were higher than the mean FPKM value. Using these criteria, we identified 687, 694, and 812 CAGs in *A. fumigatus*, *A. niger*, and *A. oryzae*, respectively. Similarly, 766, 1,241, and 749 GeAGs were identified in *A. fumigatus*, *A. niger*, and *A. oryzae*, respectively (Fig. 1).

We used freshly harvested conidia to investigate the conidial transcriptome. Thus, whether the transcriptome could change during preservation (7 d) after drying, when the resting conidia should be metabolically inactive, was of interest. To address this, we compared *A. fumigatus* transcriptomes between the freshly harvested conidia and the dried resting conidia, focusing on 687 CAGs (data not shown). In the resting conidia, only 4 and 6 % of the CAGs showed a more than fourfold increase and a less than ¼ decrease, respectively, compared with the freshly harvested conidia. Whereas 58 % of the CAGs showed an unchanged expression level, less than twofold, during preservation, and 69 % of all of the genes were unchanged in their expression levels. These results suggested that the mRNA profiles of the conidia were largely unaffected during the 7-day incubation, and specific mRNA degradation did not occur during drying.

### Functional classifications of CAGs and GeAGs using gene ontology (GO) terms

To gain a comprehensive insight into the specific molecular functions of the CAGs and GeAGs, a functional classification analysis was conducted. The total numbers of GO terms for *A. fumigatus*, *A. niger*, and *A. oryzae* were 233, 244, and 235, respectively. Among them, 8, 2, and 14 terms were significantly overrepresented or underrepresented in the CAGs, respectively (Additional file 2). The three *Aspergillus* species shared only one CAG-enriched GO term that is associated with glyoxysome (Table 2). In addition, functional classes related to adaptation to the intracellular oxidative state, such as oxidation-reduction process, oxidoreductase activity, and response to oxidative stress, were found in each fungus (Additional file 2). In the same way, 50, 70, and 59 terms were found in the GeAGs, respectively (Additional file 3). The larger number of terms found in GeAGs suggested that more molecular functions were triggered during the beginning of germination in the all three fungi. Indeed, 30 GO terms were commonly overrepresented in the GeAG of the three species (Table 2). Most were associated with ribosome function and primary cellular activities, such as translation, respiration, and metabolism, which supported the previous view that the construction of translational machinery starts at a very early stage in germination [2–4].

**Table 2:** Common CAG- and GeAG-enriched GO terms

			Count (changed: not changed)			
	GO	terms	*A. fumigatus*	*A. niger*	*A. oryzae*	Over (+)/under (−)
CAG-enriched						
# C — Cellular component						
	GO:0009514	glyoxysome	13:24	10:28	15:21	+
GeAG-enriched						
# C — Cellular component						
	GO:0005730	nucleolus	113:56	119:40	106:50	+
	GO:0030686	90S preribosome	37:0	34:1	33:1	+
	GO:0005829	cytosol	243:1105	333:986	225:1050	+
	GO:0032040	small-subunit processome	32:2	35:0	33:2	+
	GO:0005840	ribosome	44:22	57:16	44:21	+
	GO:0005762	mitochondrial large ribosomal subunit	26:4	30:0	21:4	+
	GO:0030687	preribosome, large subunit precursor	24:3	20:1	17:4	+
	GO:0005739	mitochondrion	71:304	95:297	57:315	+
	GO:0009986	cell surface	21:51	29:46	25:52	+
	GO:0031966	mitochondrial membrane	12:13	14:13	11:11	+
	GO:0005886	plasma membrane	42:224	73:191	43:214	+
	GO:0005634	nucleus	154:1289	238:1331	140:1322	+
	GO:0005819	spindle	8:13	10:12	7:13	+
	GO:0016021	integral component of membrane	15:555	36:695	17:780	-
# F — Molecular function						
	GO:0003735	structural constituent of ribosome	110:18	126:3	95:9	+
	GO:0003723	RNA binding	44:83	57:55	42:64	+
	GO:0008026	ATP-dependent helicase activity	14:25	19:22	12:27	+
	GO:0003743	translation initiation factor activity	12:16	23:6	15:12	+
	GO:0051082	unfolded protein binding	14:30	21:25	13:28	+
	GO:0005524	ATP binding	69:439	102:475	64:506	+
	GO:0003676	nucleic acid binding	51:286	76:236	49:227	+
	GO:0005525	GTP binding	18:75	24:68	21:81	+
	GO:0000166	nucleotide binding	35:208	53:208	36:244	+
	GO:0016491	oxidoreductase activity	8:391	20:634	12:635	-
	GO:0000981	sequence-specific DNA binding RNA polymerase II transcription factor activity	2:238	12:326	2:248	-
# P — Biological process						
	GO:0006412	translation	86:14	97:3	72:11	+
	GO:0006364	rRNA processing	26:7	24:4	23:9	+
	GO:0000447	endonucleolytic cleavage in ITS1 to separate SSU-rRNA from 5.8S rRNA and LSU-rRNA from tricistronic rRNA transcript (SSU-rRNA, 5.8S rRNA, LSU-rRNA)	20:2	21:1	19:2	+
	GO:0000027	ribosomal large subunit assembly	18:4	19:1	17:3	+
	GO:0035690	cellular response to drug	34:151	49:135	37:157	+
	GO:0006696	ergosterol biosynthetic process	10:16	11:15	8:19	+
	GO:0006413	translational initiation	9:12	17:5	11:9	+
	GO:0009060	aerobic respiration	9:18	12:11	9:16	+
	GO:0008152	metabolic process	9:324	16:468	6:513	-
	GO:0055085	transmembrane transport	16:405	21:552	16:651	-

### Expression profile of genes encoding allergenic proteins

*A. fumigatus* was reported to possess 22 known allergenic proteins, which is the highest number in fungi found to date [10]. A comprehensive view of the allergenic gene expression would be useful for understanding which cellular forms of the fungus were potential allergens. Our transcriptome data revealed that 14, 9, and 9 allergen-related genes showed FPKM values higher than the corresponding mean FPKM in hyphae, conidia, and 1 h-grown conidia, respectively (Additional file 4). Among the highly expressed genes, 8 genes were commonly found in all cellular forms, 5 transcripts (*aspf3*, *aspf8*, *cyp4*, *hsp90*, and *rpL3*) of which were most abundant in 1 h-grown conidia. Notably, previous proteome research revealed that AspF3 and AspF8 were presented in the *A. fumigatus* germinating conidia [11]. In addition, the other *A. fumigatus* allergenic genes (Asp f1, Asp f4, sod3, and Asp f7) were highly expressed in hyphae but relatively silent in conidia. These transcriptional data suggest that higher amount of allergenic proteins may be present in hyphae and germinating conidia of *A. fumigatus*.

### Expression profiles of common orthologous genes

In addition to species-specific allergenic genes, the *Aspergillus* species should share a large number of genes that are involved in common biological functions, such as primary metabolism and biosynthesis for cellular components. To gain more general insights into *Aspergillus* biology, we focused on the sets of orthologous genes that were found in each genome of *A. fumigatus*, *A. niger*, and *A. oryzae* and that had the highest homology levels. Using the three homology searches, *A. fumigatus* vs. *A. niger*, *A. fumigatus* vs. *A. oryzae*, and *A. nige*r vs. *A. oryzae*, we found 6,172 sets of common genes whose counterparts represented 63.1, 43.9, and 51.9 % of all of the genes in *A. fumigatus*, *A. niger*, and *A. oryzae*, respectively (Additional file 5). The RNA-sequencing data showed that 5,972 (96.3 %), 5,758 (93.3 %), and 5,636 (91.3 %) of the common genes were expressed in *A. fumigatus*, *A. niger*, and *A. oryzae*, respectively.

**Fig. 2:**
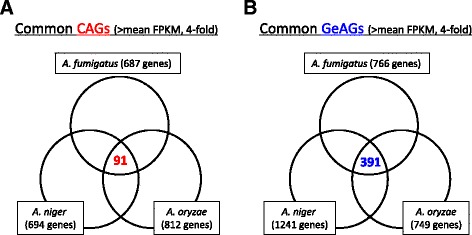
Venn diagrams comparing the CAGs and GeAGs from the three *Aspergillus* species. The common CAGs (**a**) and common GeAGs (**b**) were identified

We next sought to identify the common genes in the CAGs and GeAGs. Among the CAGs of the three species, 91 genes were found to be common, which was only 11.2–13.2 % of the CAGs in each species (Fig. 2a). These ratios were lower than those for whole gene sets in each species (43.9–63.1 %), suggesting that the CAGs are more diverse and strain specific compared with the non-CAGs. Of the 91 genes, 43 were greater than 10-fold the FPKM ratio of conidia to hyphae in all of the species (Table 3). While there was no information on 17 of the genes’ encoded protein functions, the rest included several genes that were previously reported to be conidia-specific or expressed during the asexual developmental stage. CatA is a conidia-specific catalase and is responsible for the stress tolerance of conidia, which was well documented in *A. fumigatus* and *A. oryzae*, as well as *A. nidulans* [12–14]. ConJ is a conidia-specific protein that was studied in *A. nidulans* [15], and was reported to be expressed during asexual development in *A. fumigatus* [16]. VosA plays an important role in conidia formation and trehalose accumulation in conidia, as shown in *A. fumigatus* and *A. nidulans* [17, 18]. PilB is enriched in the conidia of *A. fumigatus* [5], and *fhk1*, encoding a hybrid histidine kinase, was reported to be up-regulated during asexual development in *A. fumigatus* [16]. These results suggested that most of the common CAGs were involved in conidia-specific functions and played an essential role in the conidial biology of *Aspergillus* species.

**Table 3:** A list of common CAGs (Ratio > 10)

			FPKM ratio of conidia to hyphae*1					
*A. fumigatus*	*A. niger*	*A. oryzae*	*A. fumigatus*	*A. niger*	*A. oryzae*	atfA-dependency *2	Gene name in Af	Annotation in *A. fumigatus*
Afu2g00200	An12g10720	AO090113000153	-	458.5	-	full	*cat3*	Catalase, putative
Afu8g01530	An12g10710	AO090113000154	1751.2	1334.9	2307.7	n.d.	*-*	HHE domain protein
Afu1g01490	An15g07300	AO090102000259	1291.8	1040.6	467.4	n.d.	*-*	Hypothetical protein
Afu2g14330	An17g01885	AO090009000665	1014.6	238.2	780.3	n.d.	*-*	Hypothetical protein
Afu6g03210	An12g10240	AO090011000656	798.1	439.3	1367.5	full	*conJ*	Conidiation-specific protein 10
Afu8g05810	An15g04670	AO090005000570	584.1	1287.1	28.3	n.d.	*-*	DUF1295 domain protein
Afu5g10160	An14g04530	AO090010000533	560.4	133.2	22.4	n.d.	*-*	ActVA 4 protein
Afu8g00600	An03g00920	AO090010000696	525.8	-	-	n.d.	*-*	Conserved hypothetical protein
Afu3g01210	An01g10950	AO090026000081	454.0	867.9	583.9	n.d.	*-*	ThiJ/PfpI family protein
Afu5g01160	An03g02190	AO090020000259	338.5	21.7	12.7	full	*-*	Monosaccharide transporter
Afu8g06020	An15g04770	AO090005000539	275.9	74.8	297.8	full	*-*	Glutamate decarboxylase
Afu6g03890	An09g03130	AO090701000158	249.3	350.6	194.9	full	*catA*	Catalase A
Afu1g03580	An18g04120	AO090009000418	196.6	288.5	629.2	n.d.	*-*	Hypothetical protein
Afu3g00640	An11g08160	AO090009000116	165.1	189.5	25.2	n.d.	*-*	Peptidoglycan binding domain protein
Afu2g01590	An03g04860	AO090701000790	125.5	22.0	74.4	full	*nce102*	Non-classical export protein (Nce2), putative
Afu2g15740	An15g05990	AO090012000251	103.7	261.2	-	full	*-*	Oxidoreductase, short chaindehydrogenase/reductase family
Afu5g03930	An09g06270	AO090102000598	100.6	220.2	70.0	partial	*-*	Alcohol dehydrogenase, putative
Afu1g13530	An08g06600	AO090012000522	100.5	74.2	35.6	n.d.	*-*	Hypothetical protein
Afu3g03940	An01g00280	AO090124000046	91.1	24.5	47.6	full	*-*	2,3-diketo-5-methylthio-1-phosphopentanephosphatase, putative
Afu3g11550	An02g07350	AO090003000710	88.6	42.0	360.8	n.d.	*-*	LEA domain protein
Afu5g03269	An09g05520	AO090102000529	88.6	54.5	72.9	n.d.	*-*	Unknown
Afu6g08320	An11g01750	AO090003000094	87.1	81.9	134.4	n.d.	*pilB*	Putative conserved eisosome component
Afu3g12760	An02g08740	AO090012000846	81.3	85.1	268.0	n.d.	*-*	Hypothetical protein
Afu4g03390	An14g02450	AO090011000203	79.2	15.6	21.0	full	*-*	Aquaporin
Afu5g13100	An14g06050	AO090120000435	77.1	16.0	78.7	n.d.	*-*	Hypothetical protein
Afu6g13470	An08g03030	AO090020000658	60.5	-	496.2	n.d.	*-*	Conserved hypothetical protein
Afu1g13550	An08g06620	AO090012000521	45.4	97.8	113.2	n.d.	*-*	Hypothetical protein
Afu5g14310	An09g01150	AO090020000287	43.8	137.2	13.0	not CAG	*-*	Short chain dehydrogenase/reductase familyprotein
Afu3g10480	An16g04420	AO090003000594	31.5	76.5	134.2	n.d.	*-*	Conserved hypothetical protein
Afu6g08730	An11g06120	AO090001000547	30.2	49.1	699.1	not CAG	*-*	6-phosphogluconate dehydrogenase,decarboxylating
Afu4g09250	An04g04280	AO090023000611	29.7	11.6	18.2	n.d.	*-*	Hypothetical protein
Afu4g05900	An04g00100	AO090023001009	25.1	35.1	24.7	n.d.	*-*	Conserved hypothetical protein
Afu4g01020	An18g01860	AO090003001579	24.1	28.6	99.1	n.d.	*fhk1*	Sensor histidine kinase/response regulator, putative
Afu6g13860	An08g08500	AO090103000362	23.9	370.9	429.6	n.d.	*-*	Conserved hypothetical protein
Afu5g09180	An07g03930	AO090020000514	23.7	671.7	448.4	n.d.	*-*	Hypothetical protein
Afu4g10860	An04g05790	AO090003001124	23.2	22.7	24.9	n.d.	*vosA*	Velvet family protein
Afu5g01290	An14g07380	AO090010000635	22.4	680.6	42.7	partial	*-*	Oxidoreductase, zinc-binding dehydrogenasefamily, putative
Afu1g03090	An01g05320	AO090005000810	21.7	17.9	16.1	n.d.	*-*	Conserved hypothetical protein
Afu2g04200	An11g02200	AO090003000208	20.8	16.6	37.7	not CAG	*hppD*	4-hydroxyphenylpyruvate dioxygenase
Afu7g01430	An12g01460	AO090010000221	16.5	109.6	37.5	full	*-*	Opsin 1
Afu2g16930	An04g09030	AO090102000125	14.8	33.2	103.7	not CAG	*-*	Succinate:fumarate antiporter (Acr1), putative
Afu2g10020	An16g05030	AO090011000634	10.1	1755.9	87.7	n.d.	*-*	Hypothetical protein
Afu2g02310	An07g06530	AO090011000512	10.0	19.7	71.1	not CAG	*sur7*	sur7 protein, putative

*1: “-”, not expressed in hyphae

*2: AtfA-dependency is clarified by real-time RT PCR. n.d., not determined

**Fig. 3:**
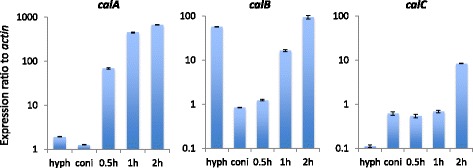
Quantitative real-time RT-PCR analyses of *calA*-family genes in *A. fumgiatus*. Expression profiles of *calA*, *calB*, and *calC* during the germination process (~2 h) and hyphae (hyph) were analyzed. Each value represents the expression ratio relative to that of the *actin* gene. Data presented are the averages of three replicates, and the bar indicates standard deviation

Likewise, we found 391 common genes in the GeAGs of the three species (Fig. 2b). This corresponded to 31.5–52.2 % of the GeAGs, and 46 of them showed more than 10-fold the FPKM ratio in 1 h-grown conidia compared with conidia in all of the species (Table 4). There were 13 genes that encoded a putative ribosome protein or a ribosome-related protein, which supported the view that *de novo* ribosome-complex biosynthesis begins at an early stage of germination. Notably, *A. fumigatus calA* (Afu3g09690) and the corresponding genes of *A. niger* (An16g03330) and *A. oryzae* (AO090005001280) showed quite high expression levels in 1 h-grown conidia (Table 4). The verification of the expression profiles during 2 h of germination using real-time PCR revealed that the *A. fumigatus calA* expression level was strongly induced at an early stage of germination (Fig. 3), suggesting that the CalA protein functions during the *A. fumigatus* germination process. Although the detailed function of CalA in germination remains unclear, CalA was detected on the swollen conidial surface by the binding of anti-CalA serum, and recombinant CalA protein was demonstrated to bind with laminin and murine lung cells [19]. CalA is a thaumatin-like protein, and *A. fumigatus* has the paralogous proteins encoded by Afu8g01710 and Afu3g00510 (*calB* and *calC*, respectively) (Additional file 6). Interestingly, the expression levels of *calB* and *calC* were induced during germination as well, while the expression level of *calB* was markedly high in hyphae (Fig. 3). In *A. niger* and *A. oryzae*, only a single protein, AoCalA and AnCalA, respectively, with high homology to the thaumatin-like protein was found, and the expression levels were also up-regulated in 1 h-grown conidia (Table 4, Additional file 6). Notably, *A. nidulans* has two thaumatin-like proteins, CetA and CalA, and the deletion of both genes was reported to result in synthetic lethality [20], suggesting an important role of the thaumatin-like proteins in *Aspergillus* conidial germination. The genetic distribution among the *Aspergillus* species (Additional file 6) suggested that the germination-related thaumatin-like protein is duplicated in some species and that their roles might be indispensable for filamentous fungi.

**Table 4:** A list of common GeAGs (Ratio > 10)

			FPKM ratio of 1 h-grown conidia to conidia				
*A. fumigatus*	*A. niger*	*A. oryzae*	*A. fumigatus*	*A. niger*	*A. oryzae*	Gene name in Af	Annotation in *A. fumigatus*
Afu3g09690	An16g03330	AO090005001280	2140.3	7365.7	1063.5	*-*	Extracellular thaumatin domain protein, putative
Afu4g08110	An04g02550	AO090023000758	75.3	163.0	12.4	*-*	Translation elongation factor G1, putative
Afu4g07630	An04g02000	AO090023000812	53.9	74.1	11.6	*-*	Microtubule associated protein (Ytm1), putative
Afu6g02690	An12g08230	AO090120000155	48.5	87.3	16.2	*mtfA*	C2H2 finger domain protein, putative
Afu1g10990	An08g03290	AO090038000305	43.2	729.2	13.4	*-*	Ribosomal RNA processing protein, putative
Afu1g04370	An01g03230	AO090003000912	39.7	2627.6	18.7	*-*	Hypothetical protein
Afu1g05310	An01g04590	AO090003000828	39.1	194.0	13.0	*-*	DUF699 ATPase, putative
Afu2g10070	An16g04970	AO090011000630	35.6	11.6	26.2	*-*	Carbamoyl-phosphate synthase, large subunit
Afu4g07540	An04g01900	AO090023000821	35.1	42.8	11.6	*-*	Small nucleolar ribonucleoprotein complexsubunit, putative
Afu2g16040	An15g06390	AO090012000197	30.2	73.6	10.6	*-*	rRNA biogenesis protein, putative
Afu2g09860	An16g05290	AO090011000649	29.0	71.4	33.7	*-*	Purine-cytosine permease
Afu1g10440	An08g02390	AO090038000383	25.7	100.8	10.2	*-*	Conserved hypothetical protein
Afu7g04700	An13g01230	AO090005000164	25.5	11.8	28.7	*-*	Conserved hypothetical portein
Afu5g03870	An09g06310	AO090102000594	23.7	42.9	10.7	*-*	Conserved hypothetical protein
Afu4g06350	An04g00680	AO090023000955	23.6	130.8	10.3	*-*	rna binding protein
Afu3g06320	An11g10000	AO090020000118	21.3	29.8	14.0	*-*	Conserved hypothetical protein
Afu2g02190	An07g06640	AO090011000497	21.0	52.7	28.0	*-*	Hypothetical nuclear protein
Afu6g08720	An11g06110	AO090001000546	20.3	38.3	23.7	*-*	5’-methylthioadenosine phosphorylase
Afu4g03650	An14g01560	AO090011000292	20.1	68.9	12.4	*-*	Ribosome associated DnaJ chaperone Zuotin, putative
Afu6g13370	An08g09160	AO090003000462	17.9	10.0	11.3	*utp10*	SSU processome component (Utp10), putative
Afu2g05950	An02g14340	AO090001000712	17.2	28.2	14.7	*-*	snRNP and snoRNP protein (Snu13), putative
Afu3g13320	An02g09200	AO090012000801	17.0	14.3	10.7	*rps0*	40S ribosomal protein S0, putative
Afu1g15730	An01g14080	AO090005001132	16.9	31.5	10.4	*-*	Ribosomal protein S8
Afu2g17200	An04g09270	AO090102000099	16.8	26.2	11.1	*-*	Hypothetical protein
Afu2g11810	An02g03860	AO090026000462	16.7	77.0	16.8	*-*	Conserved hypothetical protein
Afu5g11470	An18g04470	AO090026000687	16.0	83.7	24.6	*-*	MYB DNA binding protein (Tbf1), putative
Afu5g06430	An17g01270	AO090009000609	15.7	76.2	10.5	*-*	Mitochondrial large ribosomal subunit L7, putative
Afu2g02150	An07g06760	AO090011000490	15.5	20.0	12.7	*S10a*	Ribosomal protein S10
Afu6g04330	An15g01160	AO090701000118	15.1	35.8	12.0	*-*	DEAH-box RNA helicase (Dhr1), putative
Afu1g05390	An18g04220	AO090009000405	14.8	28.8	10.6	*-*	Mitochondrial ADP,ATP carrier protein (Ant), putative
Afu1g11710	An08g03910	AO090038000249	13.9	16.2	13.2	*-*	60S ribosomal protein L1
Afu8g05330	An16g07400	AO090005000616	13.9	16.1	10.8	*-*	Methylenetetrahydrofolate dehydrogenase
Afu3g10800	An02g06530	AO090003000629	13.7	10.4	10.9	*-*	Eukaryotic translation initiation factor 3subunit CLU1/TIF31, putative
Afu3g11260	An02g07010	AO090003000679	13.6	37.3	16.5	*ubiC*	Ubiquitin (UbiC), putative
Afu3g12300	An02g08080	AO090005000737	13.4	20.5	11.2	*-*	Ribosomal L22e protein family
Afu5g12360	An14g06860	AO090120000354	13.3	15.2	12.8	*-*	Mitochondrial oxaloacetate transporter (Oac),putative
Afu3g06580	An11g09740	AO090020000342	13.1	38.1	14.1	*-*	WD repeat protein
Afu3g05490	An11g11150	AO090020000020	13.0	72.7	13.3	*-*	Nrap protein superfamily
Afu6g10460	An11g00990	AO090023000242	12.8	10.8	15.5	*lag1*	Ceramide synthase membrane component (Lag1),putative
Afu6g09990	An11g04985	AO090038000466	12.1	29.5	10.7	*-*	Importin beta-4 subunit, putative
Afu6g08580	An11g05510	AO090001000519	11.9	16.0	10.4	*fkbp4*	FKBP-type peptidyl-prolyl isomerase, putative
Afu3g10660	An02g06320	AO090003000611	11.5	23.0	47.8	*erg13*	Hydroxymethylglutaryl-CoA synthase
Afu4g13170	An01g08850	AO090009000264	11.2	23.6	16.9	*cpcB*	Guanine nucleotide-binding protein subunit, putative
Afu3g00880	An09g03120	AO090003001496	11.1	486.0	48.1	*-*	Conserved hypothetical protein
Afu6g09060	An11g06810	AO090001000583	10.7	50.8	11.3	*-*	Mitochondrial 60S ribosomal protein L6precursor
Afu2g03380	An07g07840	AO090120000249	10.6	15.7	11.2	*-*	Alkaline serine protease

### Role of the AtfA transcription factor in regulating CAGs

**Fig. 4:**
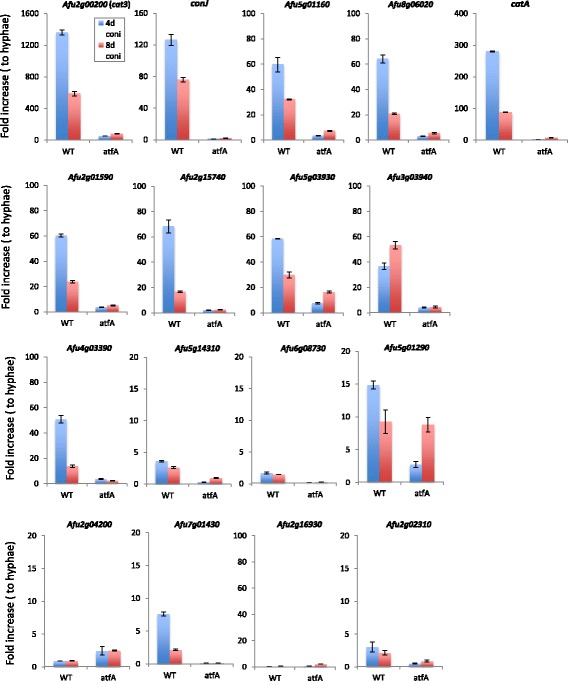
Quantitative real-time RT-PCR analyses of the common CAGs in *A. fumgiatus*. Expression levels of the common CAGs in 4- and 8-day-old conidia of Afs35 (WT) and *ΔatfA* were analyzed. Each value represents a fold increase in the expression ratios compared with those in hyphae. Data presented are averages of three replicates, and the bar indicates standard deviation

In our previous study, we demonstrated that *A. fumigatus catA*, *conJ*, and *fhk1* were up-regulated during the asexual stage in an AtfA-dependent manner [16]. AtfA is a bZip-type transcription factor that is involved in conidial stress tolerance, and the molecular function of AtfA is largely conserved in some *Aspergillus* species [16, 21, 22]. As described above, the known AtfA-dependent genes were commonly enriched in the conidia of *A. oryzae*, *A. niger*, and *A. fumigatus* (Table 3). Thus, it was of interest to investigate whether conidia-accumulated transcripts of common CAGs were dependent on the AtfA transcription factor. We cultivated the *A. fumigatus* control strain (AfS35) and *atfA* deletion mutant, and the expression levels of the selected genes in hyphae and 4- and 8-day-old conidia were analyzed using quantitative real-time RT-PCR. Most of the CAGs tested showed AtfA-dependent expression in resting conidia (Fig. 4). This clearly indicated that AtfA played a major role in conidial biology.

**Fig. 5:**
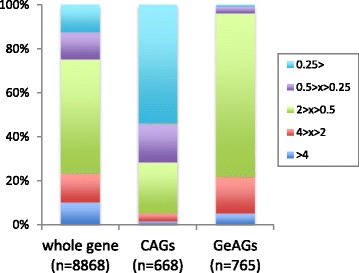
Distribution of genes with differential expression level in the *atfA* mutant conidia. Comparison between conidia of *A. fumgiatus* Afs35 (WT) and *ΔatfA*. From the FPKM values, the relative expression ratios of all of the genes (left), the CAGs (middle), and the GeAGs (right) were calculated as *ΔatfA*/WT conidia

**Fig. 6:**
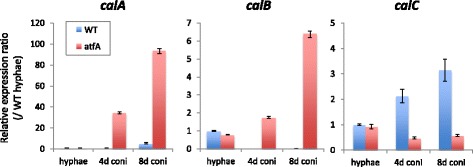
Expression levels of *calA*, *calB*, and *calC* in 4- and 8-day-old conidia of Afs35 (WT) and *ΔatfA* were analyzed. Each value represents the expression ratios compared with those in WT hyphae. Data presented are averages of three replicates, and the bar indicates standard deviation

In our previous study, the deletion of AtfA resulted in stress-labile conidia and delayed germ-tube formation [16]. To gain further insight into role of AtfA in conidial longevity, conidial transcriptomes were compared between the wildtype and the *atfA* mutant (Fig. 5). Among the whole genes, 10 and 13 % showed increased (>4 times) and decreased (< ¼ times) expression levels, respectively, in conidia of the *atfA* deletion mutant. For the CAGs, 54 % showed a decreased level (more than fourfold) of expression in the *atfA* mutant conidia compared with the wildtype. For the common CAGs, 63 % had lower expression levels in the *A. fumigatus atfA* mutant conidia (data not shown), which possibly led to deleterious defects in stress-homeostasis. Unexpectedly, a portion of GeAGs showed higher expression levels in the conidia of the *atfA* mutant. Particularly, *calA* and *calB* were highly up-regulated in the *atfA* conidia (Fig. 6). This derepression of the germination-associated genes in the resting conidia suggested that uncontrolled exit from dormancy might occur in the conidia.

### *Conidia of the null* atfA *mutant revealed germination-related traits*

**Fig. 7:**
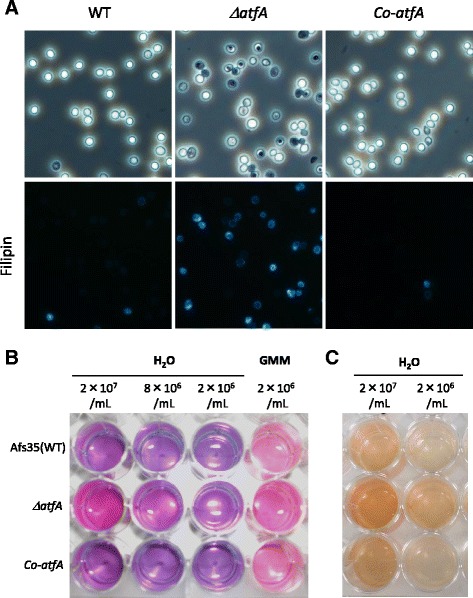
Metabolic activities in *ΔatfA* resting conidia. **a** Ergosterols were stained by filipin in the resting conidia of *A. fumigatus* Afs35 (WT), *ΔatfA*, and the complemented strain (*Co-atfA*). The test was repeated twice, and more than three sections were observed. The representative photos were shown. **b** The resazurin assay indicates metabolic activity in the *ΔatfA* resting conidia, which are without nutrients. **c** The XTT assay shows metabolic activity in the *ΔatfA* resting conidia, which are without nutrients

The first step toward germination, after sensing available carbon sources and water, is isotropic swelling, which is followed by polarized growth with germ tube formation [23]. During the isotropic growth, *de novo* ergosterol biosynthesis begins and a sterol-rich domain is observed in a polar position of the swollen conidia [24]. To investigate if conidia defective in the *atfA* gene showed precocious swelling, the freshly harvested conidia of the *atfA* mutant were microscopically observed by staining with filipin, which binds to ergosterol. Most of the conidia of the *atfA* mutant, but not of wildtype, were stained with filipin (Fig. 7a). Notably, some *atfA* mutant conidia showed sterol-rich domain formation and filipin staining at putative organelle membranes (Fig. 7a). These results supported the idea that resting conidia lacking AtfA began germination process without any available nutrients, such as carbon sources.

We then investigated the metabolic activity of the resting conidia using respiration indicators, resazurin, and XTT reagents (Fig. 7b and c). When incubated in water without any carbon sources, the wildtype conidia showed no change in color, indicating a metabolically inactive state. On the contrary, the water containing *atfA* mutant conidia changed color based on the respiration markers, suggesting that the conidia defective in AtfA were somehow metabolically active even in the absence of nutrients. Collectively, these germination-related traits suggested that AtfA has a role in maintaining conidial dormancy, and the deletion of AtfA led to the derepression of dormancy.

## Discussion

Dormant conidia of filamentous fungi are easy-to-disperse, stress-tolerant structures [1]. These properties enable the fungi to survive in a variety of harsh environments. Thus, there is a keen interest in the molecular mechanisms of conidial dormancy and germination. We used *Aspergillus* conidia as a model to gain a transcriptomic view of dormancy and the exit from dormancy. We used three *Aspergillus* species for transcriptome comparisons, which enabled us to achieve more universal findings.

The comparative analysis identified the common CAGs and GeAGs, which revealed that transcripts associated with stress tolerance and ribosome biogenesis are abundant in the conidia and 1 h-grown conidia, respectively. In fact, CatA, Cu/Zn superoxide dismutase (*sod1*), and trehalose synthase (*tpsA*) genes were found to be highly expressed in the conidia, which was consistent with the previous studies [13, 25, 26]. The conidia devoid of CatA showed an increased sensitivity to hydrogen peroxide in *A. fumigatus* and *A. nidulans*, indicating that oxidative stress tolerance in the *Aspergillus* conidia requires CatA [12, 13]. Our transcriptome data also showed that unstudied putative catalase genes (Afu2g00200, An12g10720, and AO090113000153) were commonly up-regulated in the conidia (Table 3). The amino acid sequences of the proteins are well conserved among the *Aspergillus* species, and they have a catalase core domain (IPRO: 11614), suggesting that it may play an important role in oxidative stress adaptation in the conidia. Notably, this putative catalase (here designated as Cat3), as well as CatA, was expressed in an AtfA-dependent manner (Fig. 4). Another catalase gene, *cat1*/*catB*, showed mycelia-specific expression in *A. niger* and *A. oryzae*, but not in *A. fumigatus* in which the expression level of *cat1* (Afu3g02270) was quite low in all of the cell forms tested (Additional file 5). The bifunctional catalase-peroxidase *cat2*/*cpeA*/*catD* was highly expressed in the conidia of *A. fumigatus* (*cat2*), whereas the expression levels were quite low in *A. oryzae* (Additional file 5). Collectively, *A. fumigatus* conidia contained high transcript levels of the three catalase genes, *catA*, *cat2*, and *cat3*, which might contribute to the protection of resting conidia from oxidative stresses in the environments.

In addition to catalases, the CAGs included an array of genes associated with conidial biology. VosA*,* a velvet-family protein, is involved in trehalose accumulation in the conidia and UV-tolerance [18]. The *opsin 1* gene encodes a protein homologous to *Neurospora crassa* rhodopsin NOP-1 that is expressed in a conidiation-related manner [27]. The most recent report demonstrated that *Fusarium fujikuroi* rhodopsin was highly accumulated in the conidia produced under light conditions and the *F. fujikuroi* rhodopsin functioned as a light-dependent proton pump [28]. One could hypothesize that light-dependent biological roles are conserved across most fungal spores/conidia. We also found that aquaporin genes were specifically expressed in the conidia. Although the function of aquaporin remains to be investigated in filamentous fungi, it was demonstrated that *S. cerevisiae* aquaporin was required for normal sporulation and freeze tolerance [29, 30], from which one could hypothesize that aquaporin might play an important role in conidial maturation by allowing water outflow. Taking these results into account, our comparative transcriptome analysis provided a novel insight into the molecular mechanisms underlying conidial dormancy and maturation. However, the GeAGs mainly included genes involved in fundamental cellular processes, such as ribosome biogenesis, nucleotide biogenesis, ubiquitin, and translation factors (Table 4). This corroborated previous transcriptome and proteome studies for *Aspergillus* species [2, 4, 6, 11].

In general, comparisons between transcriptome and proteome data provide results that may aid in understanding cellular functions and mechanisms. Teutschbein et al. [5] previously presented 449 proteins from the *A. fumigatus* resting conidia. We compared the conidial proteins against the transcriptome data from the present study, revealing that 448 had transcripts in the conidia and 284 (63.4 %) exhibited no less than the mean FPKM value. The genes corresponding to the proteins from the 40 most abundant spots in the conidial proteome analysis all showed greater than average FPKM values, except *rodA*, and 21 (52.5 %) were more highly expressed in the conidia than in the hyphae (> fourfold). This suggested a good accordance between the proteins expressed and the genes present in the resting conidia. However, when we compared the 100 most highly expressed CAGs against the 448 conidial proteins, only 26 corresponding proteins were found. When we compared the 687 CAGs against the proteins, 104 of the CAGs (15.1 %) were found in the conidial proteome. This comparison suggested that the transcripts with high FPKM levels were not necessarily translated in the resting conidia and that they might exist in an mRNA form. If this hypothesis is true, the conidial dormancy (metabolic inactive state) could be resulted from the absence of proteins necessary for metabolic and cellular activities in the resting conidia. Furthermore, the pre-packed mRNAs in resting conidia could contribute to an immediate response to exit from dormancy when the conidia sense appropriate nutrients in the extracellular environment. However, there is an open question regarding the mechanism underlying the preservation of mRNA in the resting conidia during dormancy.

In the present study, more than half of the *A. fumigatus* CAGs were regulated for their expression in an AtfA-dependent manner (Fig. 5). Compared with the *A. fumigatus* CAGs, the common CAGs contained a higher percentage (64 %) of AtfA-dependent genes, indicating that AtfA plays a more crucial role in the biology of *Aspergillus* resting conidia than was previously thought. The function of AtfA has been intensely studied in *A. nidulans*, *A. oryzae*, and *A. fumigatus*, in which the deletion of the *atfA* gene commonly resulted in extremely stress-labile conidia and the down-regulation of *catA* expression in the conidia [16, 21, 22]. As stated above, our data showed that not only *catA*, but also other catalase genes were highly expressed in the conidia of *A. fumigatus* in an AtfA-dependent manner. The dehydrin-like proteins DprA, DprB, and DprC, which play roles in the stress adaptation of conidia, were also enriched in the *A. fumigatus* conidia, and the transcriptional regulation of DprA and DprC was largely dependent on AtfA (Additional file 7). Notably, the *atfA* deletion mutants of *A. fumigatus* and *A. nidulans* are able to produce as many conidia as the wildtype [16, 21]. This clearly indicated that AtfA has no role in conidiation, and suggested that AtfA is likely to be essential for the conidial maturation process, in which the conidia enter dormancy and become more tolerant to environmental stresses.

In the germination process, *calA* was highly up-regulated in all of the species tested. This suggested that CalA protein commonly play an important role in initiating the germination of *Aspergillus* conidia. In fact, it was previously reported in *A. nidulans* that CalA and the paralog protein CetA were required for normal germination and that the GFP-fused proteins were localized to the hyphal periphery [20]. Intriguingly, plant thaumatin-like proteins are able to bind to polysaccharides like beta-glucan and showed beta-1,3-glucanase activity [31–33]. Some were identified as antifungal proteins induced by pathogen exposure. From these characteristics, one could hypothesize that fungal thaumatin-like proteins are involved particularly in conidial isotropic growth (swelling) by loosening cell walls with hydrolyzing activity. This hypothesis was partly supported by the findings that the abnormal expression of *calA* and *calB* (Fig. 6) and an abnormal progression of swelling (Fig. 7a) were found in the *atfA* mutant’s resting conidia. The fungal thaumatin-like proteins were widely conserved in ascomycetes to basidiomycetes, including plant pathogens, which suggested an important role in the biology of filamentous fungi [34]. Therefore, a further functional analysis of the CalA-family protein may increase our understanding of the germination mechanism, as well as the interactions, between plants and pathogens.

One of the notable findings is the derepression of dormancy in the conidia of the *A. fumigatus atfA* deletion mutant, in which filipin-positive staining and metabolic activity, that were absent in the wildtype conidia, were found. These findings clearly indicated an abnormal exit from dormancy and the partial progression of the germination process. In general, the germination process of *Aspergillus* conidia starts when the cells perceive carbohydrates, like glucose. The nutrient cues are sensed and signaled via G-proteins and the cAMP-dependent protein kinase A signaling pathway [35, 36]. In the *atfA* mutant conidia, the signaling pathway might be constitutively activated by an unknown mechanism and subsequently the germination process starts without any extracellular nutrients. The source of the energy for boosting the metabolism in the resting conidia is unknown. One possibility is the glucose derived from the degradation of conidia-accumulated trehalose, which is thought to be used during germination in wildtype conidia. In fact, a lowered level of trehalose was found in the *atfA* deleted conidia compared with the wildtype conidia [16]. It may be possible that trehalose is abnormally degraded in the resting conidia of the *atfA* mutant, which in turn results in the exit from dormancy. Besides extracellular glucose, precocious consumption of the immobilized intracellular glucose may account for delayed germination phenotype of the *atfA* mutant. Although the detailed mechanisms are still obscure, AtfA is essential for the maintenance of metabolically inactive dormancy in resting conidia.

## Conclusions

Comparisons among transcriptomes of hyphae, conidia, and 1 h-grown conidia of *Aspergillus* species provided a wide array of genes differentially expressed in each form. These findings further highlighted the essential role of the AtfA transcription factor in conidial dormancy. In addition, the thaumatin-like proteins are exclusively expressed at the germination stage, suggesting an important role in the germination of *Aspergillus* species. These results will facilitate the further investigation of molecular mechanisms underlying conidial dormancy and germination.

## Methods

### Strains and growth conditions

*A. fumigatus* strain Af293, *A. niger* strain IFM 58835, and *A. oryzae* strain RIB40 were used for the transcriptome analysis. To investigate AtfA roles, *ΔatfA* and the corresponding parental strain Afs35 of *A. fumigatus* were used [16]. All strains were cultivated on PDA plates. To collect hyphae, the strains were cultured in PDB for 18 h (*A. fumigatus*) or 26 h (*A. niger* and *A. oryzae*).

### Conidia preparation

Conidia of each strain were stored in 20 % glycerol in a −80 °C freezer. To prepare fresh conidia, the stored conidia were inoculated on a PDA slant and incubated at 37 °C (*A. fumigatus*) or 30 °C (*A. niger* and *A. oryzae*) for 1 week. The conidia were harvested with phosphate-buffered saline (PBS) containing 0.1 % Tween 20, and the concentration was calculated by counting the conidia with a hemocytometer (Watoson, Kobe, Japan). To collect conidia for RNA purification, conidia were mixed with 15 mL of PDA (final concentration, approximately 10^4^ conidia/mL) before the medium solidified in a 100-mL flask, and they were incubated at 30 or 37 °C for 7 d in the dark. After cultivation, conidia were harvested with PBS-Tween 20, filtered through a Miracloth, counted with a hemocytometer, and washed once with PBS-Tween 20. Microscopic observations revealed that there were no hyphal fragment contaminants in the conidial suspension, which contained exclusively conidia.

When 1 h-grown conidia were collected, approximately 10^9^ conidia were shaken in 10 mL PDB for 1 h and then centrifuged. To prepare the dried conidia, freshly harvested conidia were centrifuged and the supernatant was discarded. The conidia were dried in a vacuum for 2 h, and then incubated at room temperature for 7 d.

### RNA and cDNA preparation

Mycelia, conidia, and 1 h-grown conidia from duplicate independent culture were pooled and frozen in liquid nitrogen, and total RNA was isolated using the FastRNA Pro Red Kit (MP Biomedicals, Santa Ana, CA, USA). To obtain cDNA pools from the total RNA, possible contaminating genomic DNA was removed, and reverse transcription was performed using the ReverTra Ace qPCR RT Master Mix with gDNA remover (Toyobo).

### RNA-sequencing analysis

An indexed cDNA library was prepared using the SureSelect Strand-Specific RNA Seq (Agilent Technologies) according to standard protocols. Briefly, each total RNA sample (1 μg) was enriched for mRNA using oligo(dT)-tagged beads. RNA samples were fragmented into smaller pieces and used to synthesize cDNAs. The library construction involved end repair, A-tailing, adapter ligation, and amplification. The mean length of each library was approximately 290 bp. Sequencing was performed in a single-end 60-base pair mode on a Hiseq system (Illumina). The RNA-sequencing read data was deposited to the DDBJ Sequence Read Archive under accession No. PRJDB4747.

### Expression analysis

Illumina data sets were trimmed using fastq-mcf in ea-utils (v1.1.2-484) [37], where sequencing adapters and sequences with low-quality scores (Phred score Q < 20) were removed. Cleaned reads were mapped to the genome sequence of *A. fumigatus* Af293 (29,420,142 bp; genome version: s03-m04-r31), *A. niger* CBS (33,975,768 bp; genome version: s01-m06-r19), or *A. oryzae* RIB40 (37,912,014 bp; genome version: s01-m08-r26) from AspGD (http://www.aspgd.org/) using TopHat (v2.0.4) with the default parameters [38]. FPKMs were calculated using cuffdiff in Cufflinks (v2.1.1) with default parameters [39]. Data analyses were conducted using the R programming language (https://www.r-project.org/), and cummeRbund software [40].

### GO analysis

Genes were functionally categorized using their GO information [41] obtained from AspGD, and overrepresented GO terms were identified using Fisher’s exact test. The one-tailed Fisher’s exact *p*-value corresponding to the overrepresentation of GO categories with equal to, or greater than, 20 genes was calculated based on counts in 2 × 2 contingency tables [42]. *p*-values were corrected by the false discovery rate method [43], and the threshold was set as 0.01.

### Identification of orthologous genes

BLASTP (v2.2.28+) analyses [44] querying all of the protein sequences from one *Aspergillus* species against those of the other *Aspergillus* species were conducted using BLOSUM 80. The reciprocal best-hit pairs between two species—7,302 genes between *A. fumigatus* and *A. niger*; 7,216 between *A. fumigatus* and A. oryzae, and 7,636 between *A. niger* and *A. oryzae*—were extracted. Finally, 6,172 orthologous gene sets were identified among the three species and used in this study.

### Quantitative real-time RT-PCR

Real-time RT-PCR was performed using SYBR Green detection as described previously [45]. The primer sets used in this study are listed in Additional file 8. The relative expression ratios were calculated by the comparative cycle threshold (Ct) (ΔΔCt) method. The *actin* gene was used as a normalization reference (internal control). Each sample was tested in triplicate.

### Filipin staining

The resting conidia of interest were harvested as described above. 10^8^ conidia were centrifuged and then washed once with PBS + Tween 20. They were dissolved in 200 μL of PBS + Tween20 (final concentration, 5 × 10^8^/mL). A stock solution (1 mM) of filipin (Sigma) was prepared in DMSO, and 5 μL of the solution was added to 50 μL of the conidial suspensions. After incubation at room temperature for 3 min, they were centrifuged, immediately dissolved by an equivalent volume of PBS + Tween20, and observed under a fluorescent microscope.

### Metabolic activity assay

To compare the respiration activity in conidia, a resazurin assay was conducted. The conidia (2 × 10^7^–2 × 10^6^) of each strain were incubated in 1 mL of distilled water with resazurin (final concentration, 0.1 mM) at 37 °C for 20 h and then photographed. The tetrazolium salt 2,3-bis(2-methoxy-4-nitro-5-sulfophenyl)-2H-tetrazolium-5-carbox-anilide (XTT) assay was conducted. The conidia (2 × 10^7^–2 × 10^6^) of each strain were incubated in 0.4 mL of distilled water with 0.1 mL of XTT solution, containing 1 mg/mL XTT and 50 μM menadione, at 37 °C for 4 h and then photographed.

### Ethics approval

Not applicable.

### Consent for publication

Not applicable.

### Availability of data and material

The datasets, supporting the conclusions of this article, is available in a BioProject PRJDB4747 deposited to DDBJ. All fungal strains are available from National BioResource Project of Mycology Research Center of Chiba University (https://daphne.pf.chiba-u.jp/distribution/catalog).

## Additional files

Additional file 1: Figure S1. Growth test for the strains used in this study. (A) The conidia (approximately 10^5^) of each strain were point-inoculated on PDA, and the palates were incubated for 5 d at the indicated temperatures before photographing. (B) The numbers of conidia were calculated. Conidia of each strain were mixed with 3 mL of PDA (final concentration, approximately 10^4^ conidia/mL) before the medium solidified in a well of 6-well plate, and they were incubated at indicated temperatures for 7 d in the dark. After cultivation, conidia were harvested with PBS-Tween 20, filtered through a Miracloth, and counted with a hemocytometer. The data were obtained in triplicate and the mean value was shown. An error bar indicates standard deviation. (C) Germination rate was calculated in each strain. The conidia (final concentration, 10^6^ conidia/mL) were incubated in 0.5 mL of PDB in an eppendorf tube and were shaken at 160 rpm. At each indicated time-point, the conidia were checked if the germ tube appeared. The data were obtained in at least 5 replicates and the mean value was shown. An error bar indicates standard deviation. (D) Growth in liquid culture was investigated. The conidia (final concentration, 2 × 10^5^ conidia/mL) were incubated in 30 mL of PDB in a 100 mL flask and were agitated at 160 rpm. At each indicated time-point, mycelia were harvested, frozen, and preserved in a −80 °C freezer before being analyzed. The mycelia were lyophilized and weighed. The data were obtained in triplicate and the mean value was shown. An error bar indicates standard deviation. (PPTX 2850 kb) Additional file 2: Table S1A. GO terms that were enriched in CAGs. (XLSX 11 kb) Additional file 3: Table S1B. GO terms that were enriched in GeAGs. (XLSX 56 kb) Additional file 4: Table S2. List of *A. fumigatus* allergen-related genes and their FPKM values. (XLSX 12 kb) Additional file 5: Table S3. FPKM values of the 6172 common genes. (XLSX 986 kb) Additional file 6: Figure S2.
*Aspergillus* CalA-family proteins. (A) The protein lengths and gene IDs are shown. (B) Alignment of the CalA-family protein sequences. This alignment was constructed by ClustalW. (PPTX 79 kb) Additional file 7: Figure S3. Quantitative real-time RT-PCR analyses of dehydrin-like genes in *A. fumgiatus*. Expression profiles of the *dprA, dprB,* and *dprC* in the resting conidia of Afs35 (WT) and *ΔatfA* were analyzed. Each value represents the expression ratio relative to that of *actin*. Data presented are averages of three replicates, and the bar indicates standard deviation. (PPTX 55 kb) Additional file 8: Table S4. List of primers used in this study. (XLSX 51 kb)

## Abbreviations

PDA: potato dextrose agar
BLOSUM: Blocks substitution matrix
bZIP: basic leucine zipper
CAG: conidia-associated gene
DMSO: dimethyl sulfoxide
FPKM: fragments per kilobase of transcript per million mapped reads
GeAG: germination-associated gene
GFP: green fluorescent protein
GO: gene ontology
PBS: phosphate-buffered saline
PDB: potato dextrose broth
RT-PCR: reverse transcription polymerase chain reaction
