# Supplementary material for: Comparative transcriptome analysis revealing dormant conidia and germination associated genes in Aspergillus species: an essential role for AtfA in conidial dormancy
Source: BMC Genomics. 2016 May 17;17:358. doi: 10.1186/s12864-016-2689-z (PMC4869263; doi:10.1186/s12864-016-2689-z)
Supplement: Additional file 1: Figure S1. — Growth test for the strains used in this study. (A) The conidia (approximately 105) of each strain were point-inoculated on PDA, and the palates were incubated for 5 d at the indicated temperatures before photographing. (B) The numbers of conidia were calculated. Conidia of each strain were mixed with 3 mL of PDA (final concentration, approximately 104 conidia/mL) before the medium solidified in a well of 6-well plate, and they were incubated at indicated temperatures for 7 d in the dark. After cultivation, conidia were harvested with PBS-Tween 20, filtered through a Miracloth, and counted with a hemocytometer. The data were obtained in triplicate and the mean value was shown. An error bar indicates standard deviation. (C) Germination rate was calculated in each strain. The conidia (final concentration, 106 conidia/mL) were incubated in 0.5 mL of PDB in an eppendorf tube and were shaken at 160 rpm. At each indicated time-point, the conidia were checked if the germ tube appeared. The data were obtained in at least 5 replicates and the mean value was shown. An error bar indicates standard deviation. (D) Growth in liquid culture was investigated. The conidia (final concentration, 2 × 10^5^ conidia/mL) were incubated in 30 mL of PDB in a 100 mL flask and were agitated at 160 rpm. At each indicated time-point, mycelia were harvested, frozen, and preserved in a −80 °C freezer before being analyzed. The mycelia were lyophilized and weighed. The data were obtained in triplicate and the mean value was shown. An error bar indicates standard deviation. (PPTX 2850 kb) [file 12864_2016_2689_MOESM1_ESM.pptx]

## Slide 1
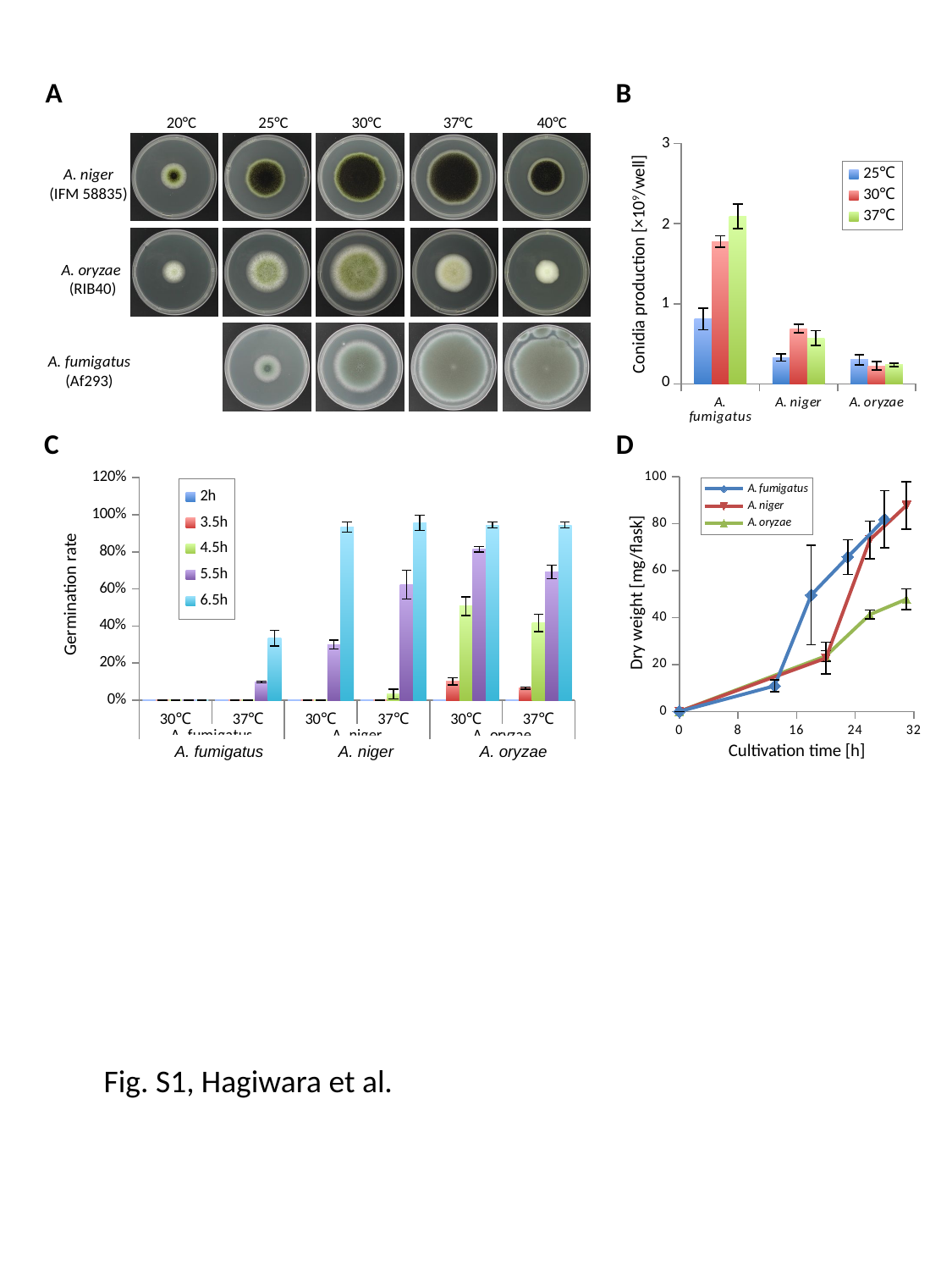

A
B
20°C
25°C
30°C
37°C
40°C
A. niger
(IFM 58835)
A. oryzae
 (RIB40)
A. fumigatus
(Af293)
3
### Chart
| Category | 25℃ | 30℃ | 37℃ |
|---|---|---|---|
| A. fumigatus | 808000000.0 | 1778666666.66667 | 2090666666.66667 |
| A. niger | 329333333.333333 | 689333333.333333 | 572000000.0 |
| A. oryzae | 301333333.333333 | 226000000.0 | 238000000.0 |2
Conidia production [×109/well]
1
0
C
D
### Chart
| Category | 2h | 3.5h | 4.5h | 5.5h | 6.5h |
|---|---|---|---|---|---|
| 30℃ | 0.0 | 0.0 | 0.0 | 0.0 | 0.0 |
| 37℃ | 0.0 | 0.0 | 0.0 | 0.0981191222570533 | 0.333066239316239 |
| 30℃ | 0.0 | 0.0 | 0.0 | 0.300198412698413 | 0.93405017921147 |
| 37℃ | 0.0 | 0.0 | 0.0329996292176492 | 0.623809523809524 | 0.95663082437276 |
| 30℃ | 0.0 | 0.101614434947768 | 0.507246376811594 | 0.81326164874552 | 0.945161290322581 |
| 37℃ | 0.0 | 0.0637292464878672 | 0.416692268305172 | 0.692063492063492 | 0.945161290322581 |Germination rate
A. fumigatus
A. niger
A. oryzae
### Chart
| Category | A. fumigatus | A. niger | A. oryzae |
|---|---|---|---|Dry weight [mg/flask]
Cultivation time [h]
Fig. S1, Hagiwara et al.
