# Supplementary material for: Comparative transcriptome analysis revealing dormant conidia and germination associated genes in Aspergillus species: an essential role for AtfA in conidial dormancy
Source: BMC Genomics. 2016 May 17;17:358. doi: 10.1186/s12864-016-2689-z (PMC4869263; doi:10.1186/s12864-016-2689-z)
Supplement: Additional file 6: Figure S2. — Aspergillus CalA-family proteins. (A) The protein lengths and gene IDs are shown. (B) Alignment of the CalA-family protein sequences. This alignment was constructed by ClustalW. (PPTX 79 kb) [file 12864_2016_2689_MOESM6_ESM.pptx]

## Slide 1
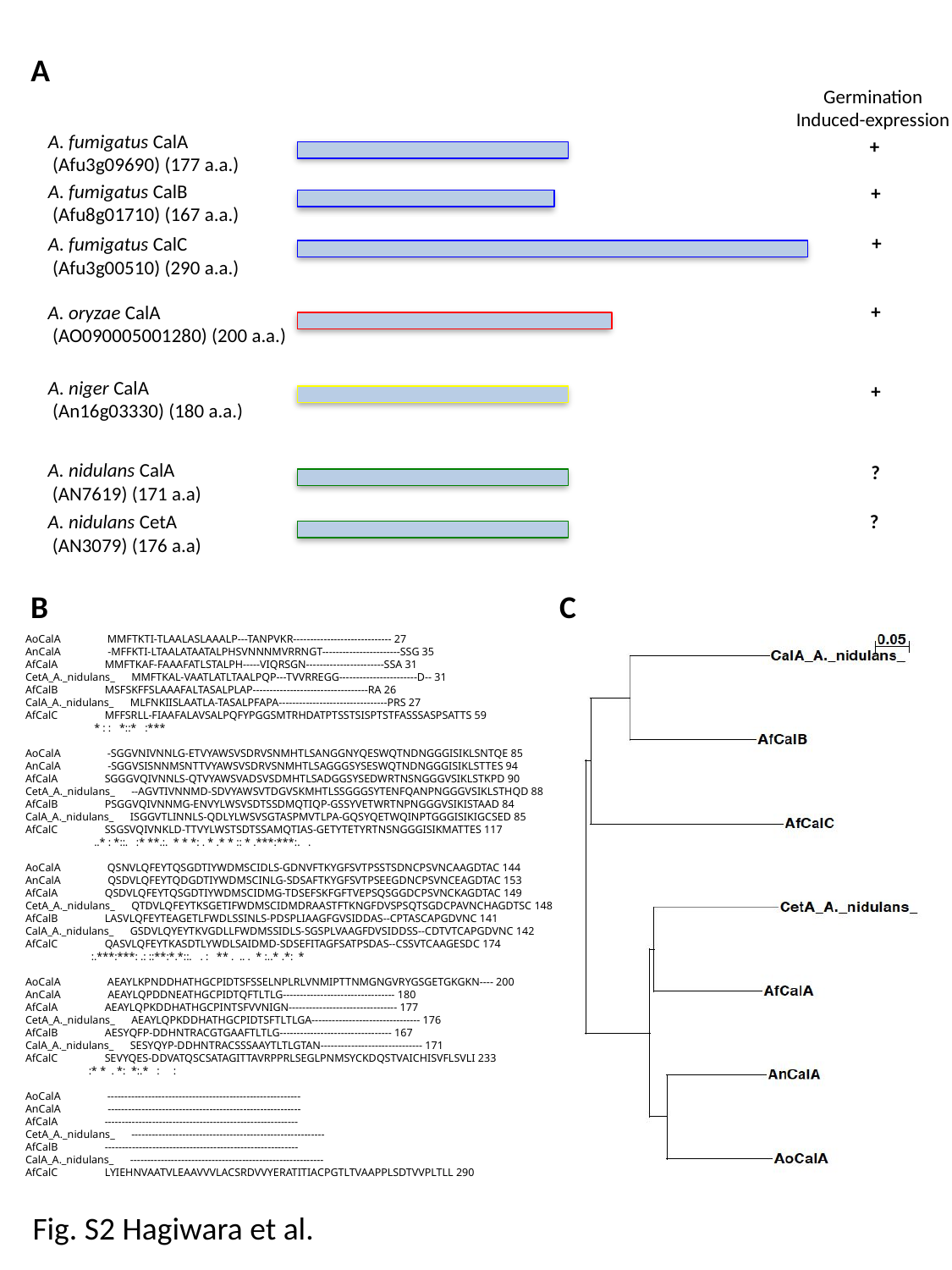

A
Germination
Induced-expression
A. fumigatus CalA
 (Afu3g09690) (177 a.a.)
+
A. fumigatus CalB
 (Afu8g01710) (167 a.a.)
+
+
A. fumigatus CalC
 (Afu3g00510) (290 a.a.)
+
A. oryzae CalA
 (AO090005001280) (200 a.a.)
A. niger CalA
 (An16g03330) (180 a.a.)
+
A. nidulans CalA
 (AN7619) (171 a.a)
?
?
A. nidulans CetA
 (AN3079) (176 a.a)
B
C
AoCalA MMFTKTI-TLAALASLAAALP---TANPVKR----------------------------- 27
AnCalA -MFFKTI-LTAALATAATALPHSVNNNMVRRNGT-----------------------SSG 35
AfCalA MMFTKAF-FAAAFATLSTALPH-----VIQRSGN-----------------------SSA 31
CetA_A._nidulans_ MMFTKAL-VAATLATLTAALPQP---TVVRREGG-----------------------D-- 31
AfCalB MSFSKFFSLAAAFALTASALPLAP----------------------------------RA 26
CalA_A._nidulans_ MLFNKIISLAATLA-TASALPFAPA--------------------------------PRS 27
AfCalC MFFSRLL-FIAAFALAVSALPQFYPGGSMTRHDATPTSSTSISPTSTFASSSASPSATTS 59
 * : : *::* :***
AoCalA -SGGVNIVNNLG-ETVYAWSVSDRVSNMHTLSANGGNYQESWQTNDNGGGISIKLSNTQE 85
AnCalA -SGGVSISNNMSNTTVYAWSVSDRVSNMHTLSAGGGSYSESWQTNDNGGGISIKLSTTES 94
AfCalA SGGGVQIVNNLS-QTVYAWSVADSVSDMHTLSADGGSYSEDWRTNSNGGGVSIKLSTKPD 90
CetA_A._nidulans_ --AGVTIVNNMD-SDVYAWSVTDGVSKMHTLSSGGGSYTENFQANPNGGGVSIKLSTHQD 88
AfCalB PSGGVQIVNNMG-ENVYLWSVSDTSSDMQTIQP-GSSYVETWRTNPNGGGVSIKISTAAD 84
CalA_A._nidulans_ ISGGVTLINNLS-QDLYLWSVSGTASPMVTLPA-GQSYQETWQINPTGGGISIKIGCSED 85
AfCalC SSGSVQIVNKLD-TTVYLWSTSDTSSAMQTIAS-GETYTETYRTNSNGGGISIKMATTES 117
 ..* : *::. :* **.:. * * *: . * .* * :: * .***:***:. .
AoCalA QSNVLQFEYTQSGDTIYWDMSCIDLS-GDNVFTKYGFSVTPSSTSDNCPSVNCAAGDTAC 144
AnCalA QSDVLQFEYTQDGDTIYWDMSCINLG-SDSAFTKYGFSVTPSEEGDNCPSVNCEAGDTAC 153
AfCalA QSDVLQFEYTQSGDTIYWDMSCIDMG-TDSEFSKFGFTVEPSQSGGDCPSVNCKAGDTAC 149
CetA_A._nidulans_ QTDVLQFEYTKSGETIFWDMSCIDMDRAASTFTKNGFDVSPSQTSGDCPAVNCHAGDTSC 148
AfCalB LASVLQFEYTEAGETLFWDLSSINLS-PDSPLIAAGFGVSIDDAS--CPTASCAPGDVNC 141
CalA_A._nidulans_ GSDVLQYEYTKVGDLLFWDMSSIDLS-SGSPLVAAGFDVSIDDSS--CDTVTCAPGDVNC 142
AfCalC QASVLQFEYTKASDTLYWDLSAIDMD-SDSEFITAGFSATPSDAS--CSSVTCAAGESDC 174
 :.***:***: .: ::**:*.*::. . : ** . .. . * :..* .*: *
AoCalA AEAYLKPNDDHATHGCPIDTSFSSELNPLRLVNMIPTTNMGNGVRYGSGETGKGKN---- 200
AnCalA AEAYLQPDDNEATHGCPIDTQFTLTLG--------------------------------- 180
AfCalA AEAYLQPKDDHATHGCPINTSFVVNIGN-------------------------------- 177
CetA_A._nidulans_ AEAYLQPKDDHATHGCPIDTSFTLTLGA-------------------------------- 176
AfCalB AESYQFP-DDHNTRACGTGAAFTLTLG--------------------------------- 167
CalA_A._nidulans_ SESYQYP-DDHNTRACSSSAAYTLTLGTAN------------------------------ 171
AfCalC SEVYQES-DDVATQSCSATAGITTAVRPPRLSEGLPNMSYCKDQSTVAICHISVFLSVLI 233
 :* * . *: *:.* : :
AoCalA ---------------------------------------------------------
AnCalA ---------------------------------------------------------
AfCalA ---------------------------------------------------------
CetA_A._nidulans_ ---------------------------------------------------------
AfCalB ---------------------------------------------------------
CalA_A._nidulans_ ---------------------------------------------------------
AfCalC LYIEHNVAATVLEAAVVVLACSRDVVYERATITIACPGTLTVAAPPLSDTVVPLTLL 290
Fig. S2 Hagiwara et al.
