# Supplementary material for: Comparative transcriptome analysis revealing dormant conidia and germination associated genes in Aspergillus species: an essential role for AtfA in conidial dormancy
Source: BMC Genomics. 2016 May 17;17:358. doi: 10.1186/s12864-016-2689-z (PMC4869263; doi:10.1186/s12864-016-2689-z)
Supplement: Additional file 7: Figure S3. — Quantitative real-time RT-PCR analyses of dehydrin-like genes in A. fumgiatus. Expression profiles of the dprA, dprB, and dprC in the resting conidia of Afs35 (WT) and ΔatfA were analyzed. Each value represents the expression ratio relative to that of actin. Data presented are averages of three replicates, and the bar indicates standard deviation. (PPTX 55 kb) [file 12864_2016_2689_MOESM7_ESM.pptx]

## Slide 1
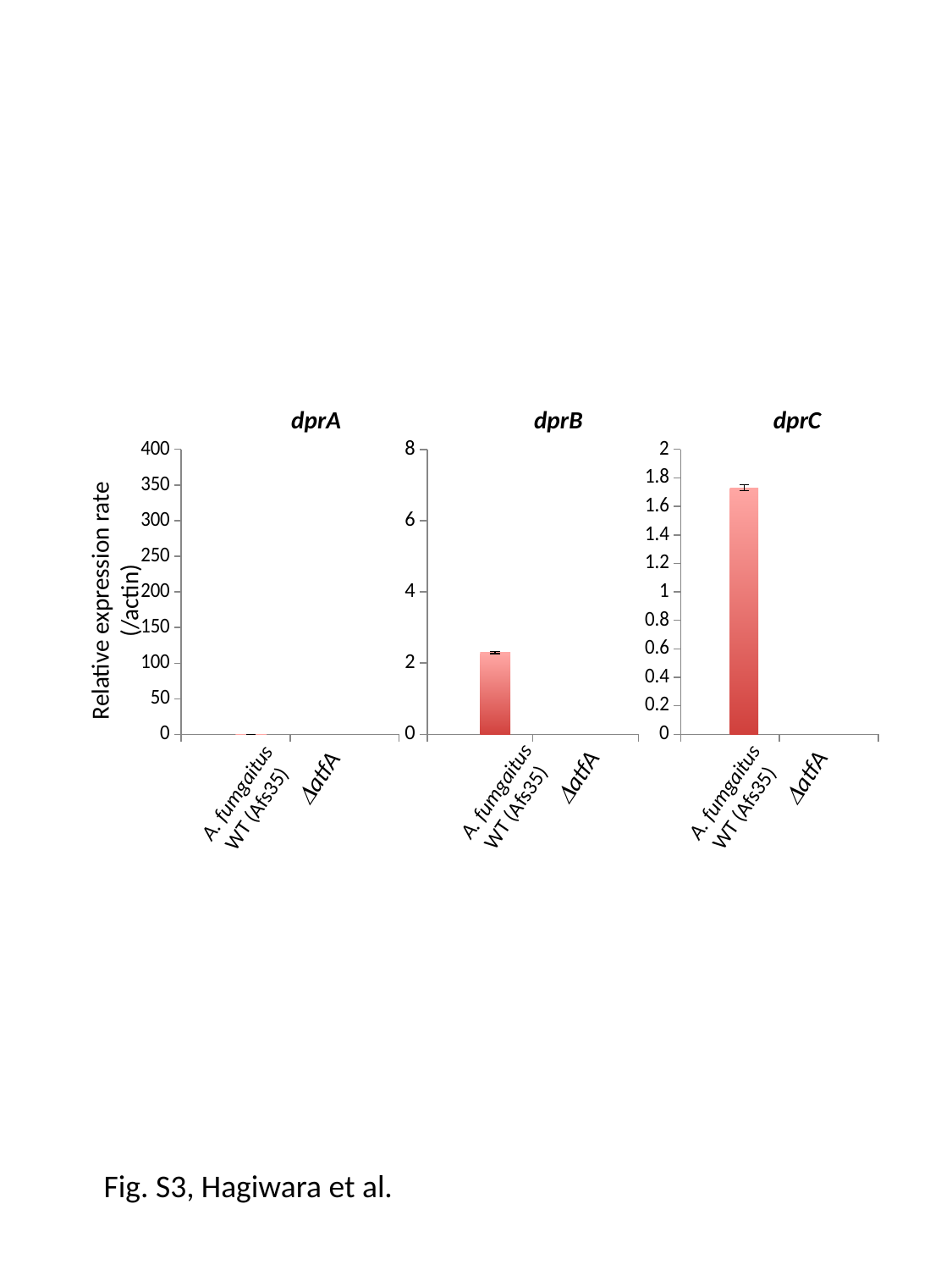

dprA
dprB
dprC
### Chart
| Category | WT | atfA |
|---|---|---|
| 37.0 | 311.2811251655937 | 0.0841593489819457 |
### Chart
| Category | WT | atfA |
|---|---|---|
| 37.0 | 5.768805403353791 | 2.296951424663638 |
### Chart
| Category | WT | atfA |
|---|---|---|
| 37.0 | 45.30273346490524 | 1.731028077505325 |Relative expression rate
(/actin)
DatfA
DatfA
DatfA
A. fumgaitus
 WT (Afs35)
A. fumgaitus
 WT (Afs35)
A. fumgaitus
 WT (Afs35)
Fig. S3, Hagiwara et al.
